# The interaction between self-care behavior and disease knowledge on the decline in renal function in chronic kidney disease

**DOI:** 10.1038/s41598-020-79873-z

**Published:** 2021-01-11

**Authors:** Yi-Chun Tsai, Shu-Li Wang, Hui-Ju Tsai, Tzu-Hui Chen, Lan-Fang Kung, Pei-Ni Hsiao, Shih-Ming Hsiao, Shang-Jyh Hwang, Hung-Chun Chen, Yi-Wen Chiu

**Affiliations:** 1 Department of Nephrology, Department of Internal Medicine, Kaohsiung Medical University Hospital, Kaohsiung Medical University, 100 TzYou 1st Road, Kaohsiung, 807 Taiwan; 2 Division of General Medicine, Department of Internal Medicine, Kaohsiung Medical University Hospital, Kaohsiung Medical University, Kaohsiung, Taiwan; 3 Faculty of Renal Care, Kaohsiung Medical University, Kaohsiung, Taiwan; 4 School of Medicine, College of Medicine, Kaohsiung Medical University, Kaohsiung, Taiwan; 5 Cohort Research Center, Kaohsiung Medical University, Kaohsiung, Taiwan; 6 Department of Nursing, Kaohsiung Medical University Hospital, Kaohsiung Medical University, Kaohsiung, Taiwan; 7 Department of Family Medicine, Kaohsiung Municipal Ta-Tung Hospital, Kaohsiung Medical University Hospital, Kaohsiung Medical University, Kaohsiung, Taiwan

**Keywords:** Health care, Nephrology

## Abstract

Multidisciplinary care can improve the outcomes of chronic kidney disease (CKD), however the contribution of self-care behavior and knowledge about CKD is unclear. This study enrolled 454 participants with CKD stages 1–5 not on dialysis. Structured questionnaires were used to evaluate self-care behavior and kidney disease knowledge. Rapid decline in renal function was defined as the decline in estimated filtration rate > 3 ml/min per 1.73 m^2^/year within 1-year prior to enrollment. The mean age of all study participants was 65.8 ± 12.1 years and 55.9% were male. The elderly had better self-care behavior while younger participants had better disease knowledge. Both high self-care and high disease knowledge scores were significantly associated with and had a synergistic effect on decreasing the risk of rapid decline in renal function. CKD patients with better self-care behavior and better kidney disease knowledge had lower risk of rapid decline in renal function.

## Introduction

Chronic kidney disease (CKD) is a global public health issue and is associated with high medical costs. Many challenges are involved in the management of CKD, including early diagnosis, prevention and the development of a thorough disease management program^1,2^. Multidisciplinary health care for CKD patients involves providing background knowledge including renal function and related clinical complication based on CKD stages of patients, and supplying appropriate treatment including medicine and lifestyle modification, such as blood pressure measurement, diet protein restriction, and avoiding nephrotoxin^3^. Increasing evidence has shown that appropriate multidisciplinary management can decrease the risks of initial dialysis and all-cause mortality^4^. Because CKD is a chronic and progressive disease, patient participation and introspection play an important role in the efficiency of multidisciplinary care programs on CKD progression^5^. In other words, to successfully retard CKD progression, patients must take an active role in the care strategy.

CKD patients have been reported as having a limited understanding of their illness^6^. Accurate disease knowledge is a key factor in self-care^7^, and this might improve clinical outcomes^8^. Self-care behavior involves self-managing the illness, but the rate of self-care among CKD patients has been reported to be low due to deficiencies in recognizing the benefits of such behavior^9^. Previous studies have reported an association between non-adherence to self-care behavior and adverse clinical outcomes in patients on dialysis^9,10^; however, the relationships among self-care behavior, disease knowledge and clinical outcomes in CKD patients not on dialysis are unclear. Therefore, the aims of this study were to analyze the factors related to self-care behavior and disease knowledge and retrospectively to examine the association of the interaction between these two components with decline in renal function (estimated glomerular infiltration rate (eGFR) slope) in patients with CKD.

## Results

### Characteristics of entire cohort

The median scores of self-care behavior and disease knowledge were 67 and 22 respectively. Comparisons of clinical characteristics among groups according to the median scores of self-care behavior and disease knowledge are shown in Table 1. High self-care behavior and high disease knowledge were defined as scores ≥ 67 and 22 respectively, while low self-care behavior and low disease knowledge were defined as scores < 67 and 22 respectively. The mean age of all participants was 65.8 ± 12.1 years (median: 66.9 years), 254 (55.9%) were male, and 382 (84.1%), 172 (37.9%), and 87 (19.2%) had hypertension, diabetes, and cardiovascular disease respectively. Three hundred and forty-eight (76.7%) patients were married, 243 (53.5%) had graduated from senior high school or above, 130 (28.6%) were currently working, and 273 (60.1%) had financial self-support. The cause of CKD included glomerulonephritis (44.3%), diabetes mellitus (DM) (22.7%), hypertension (16.3%), polycystic kidney disease (4.0%), gout/stone (8.8%), and others (4.0). There were no significant differences in patients stratified by the median scores of self-care behavior and disease knowledge. Among the four groups, patients with high self-care behavior and high disease knowledge had the highest proportion of education with high school or above, the longest CKD duration and health education times, and the lowest proportion of smoking and DM. Patients with low self-care behavior and high disease knowledge were youngest, had the highest proportion of working currently and financial self-support. Patients with high self-care behavior and low disease knowledge were the oldest, had the lowest proportion of working currently and education with high school or above. Patients with low self-care behavior and low disease knowledge had the lowest proportion of financial self-support and the highest proportion of DM.

**Table 1:** The clinical characteristics of study patients stratified by the medians levels of self-care behavior and disease knowledge.

	Entire cohort N = 454	Low self-care score and low disease knowledge score N = 133	High self-care score and low disease knowledge score N = 95	Low self-care score and high disease knowledge score N = 99	High self-care score and high disease knowledge score N = 127	p-value
**Demographics**						
Age (year)	65.8 ± 12.1	66.6 ± 11.8^#&^	71.8 ± 9.0*^&^	59.3 ± 12.7*^#^	65.5 ± 11.7^#&^	< 0.001
Sex (male, %)	55.9	52.6	54.7	64.6	53.5	0.3
Smoking (yes, %)	22.2	26.3	25.3	27.3	11.8*^&^	0.01
Alcohol consumption (yes, %)	10.4	11.3	9.5	12.1	8.7	0.8
Marital status (yes, %)	76.7	75.2	73.7	72.7	83.5	0.2
Currently working (yes, %)	28.6	27.8^&^	12.6^&^	48.5*^#^	26.0^&^	< 0.001
Independent finances (yes, %)	60.1	48.9^&^	55.8	68.7*	68.5*	0.002
Education (high school or above, %)	53.5	39.8^&^	36.8^&^	65.7*^#^	70.9*^#^	< 0.001
CKD cause (yes, %)						0.1
Glomerulonephritis	44.3	35.3	43.2	44.4	54.3	
Diabetes mellitus	22.7	33.8	24.2	19.2	12.6	
Hypertension	16.3	15.0	17.9	17.2	15.7	
Polycystic kidney disease	4.0	3.0	3.2	4.0	5.5	
Gout/stone	8.8	9.0	7.4	11.1	7.9	
Others	4.0	3.8	4.2	4.0	3.9	
**Comorbidities**						
Hypertension (yes, %)	84.1	78.9	89.5	84.8	85.0	0.2
Diabetes mellitus (yes, %)	37.9	42.9	48.4	37.4	25.2*^#^	0.002
Heart disease (yes, %)	19.2	20.3	25.3	19.2	13.4	0.2
**Clinical characteristics**						
Body mass index (kg/m^2^)	24.7 ± 4.3	25.2 ± 4.2	24.5 ± 3.5	25.4 ± 4.8	23.9 ± 4.5	0.02
ACEI/ARB usage (%)	31.9	28.6	30.5	34.3	34.6	0.7
Health education times	15.4 ± 10.4	13.25 ± 9.9^&^	15.6 ± 10.9	15.0 ± 10.5	17.8 ± 10.1*	0.005
CKD duration (year)	9.5 ± 7.6	7.4 ± 5.9^&^	8.7 ± 7.2	10.5 ± 8.4*	11.3 ± 8.3*	< 0.001
CKD care duration (year)	5.0 ± 3.7	4.1 ± 3.4^&^	5.3 ± 4.1	5.0 ± 3.6	5.6 ± 3.6*	0.005
**Questionnaires**						
Self-care score	64.1 ± 9.7	55.3 ± 7.0^#&^	71.6 ± 3.9*^&^	58.0 ± 6.7*^#^	72.5 ± 4.0*^&^	< 0.001
Disease knowledge score	22.7 ± 5.6	18.1 ± 3.0^&^	18.5 ± 2.9^&^	26.7 ± 3.8*^#^	27.7 ± 3.9*^#^	< 0.001
**Laboratory parameters**						
Blood urea nitrogen (mg/dl)	27.8 (19.8, 43.9)	29.6 (22.4, 44.5)	28.6 (19.4, 39.3)	23.2 (6.7, 38.8)	31.0 (19.8, 55.8)^# &^	0.004
eGFR (ml/min/1.73 m^2^)	34.6 ± 23.0	32.2 ± 22.6^&^	35.5 ± 19.0	42.1 ± 26.6*	30.5 ± 21.9^&^	0.001
Glycated hemoglobin (%)	5.9 (5.5, 6.5)	6.1 (5.6, 7.0)	5.9 (5.6, 6.5)	5.9 (5.5,6.5)	5.8 (5.5, 6.2)*	0.03
Hemoglobin (g/dl)	11.9 ± 2.0	11.8 ± 2.0^&^	11.9 ± 1.8	12.6 ± 2.1*	11.7 ± 2.0^&^	0.005
Albumin (g/dl)	4.3 ± 0.4	4.2 ± 0.4	4.3 ± 0.3	4.3 ± 0.3	4.3 ± 0.4	0.3
Uric acid (mg/dl)	6.6 ± 1.6	6.7 ± 1.5	6.5 ± 1.7	6.5 ± 1.6	6.6 ± 1.4	0.7
Cholesterol (mg/dl)	174 ± 37	174 ± 39	173 ± 36	176 ± 44	173 ± 33	0.9
Triglyceride (mg/dl)	108 (79, 163)	112 (79, 172)	107 (79, 150)	122 (82, 180)	102 (73, 145)	0.09
Urine protein/creatinine ratio	0.6 (0.2, 1.5)	0.6 (0.2, 1.6)	0.5 (0.2, 1.4)	0.4 (0.1, 1.2)	0.6 (0.2, 1.4)	0.4

Data are expressed as number (percentage) for categorical variables and mean ± SD or median (25th, 75th percentile) for continuous variables, as appropriate.

ACEI/ARB, angiotensin converting enzyme inhibitors/angiotensin II receptor blockers; eGFR, estimated glomerular filtration rate.

Low self-care as less than median of self-care score (67); High self-care as above median of self-care score (67).

Low disease knowledge as less than median of disease knowledge score (22); High disease knowledge as above median of disease knowledge score (22).

The missing data included serum albumin (n = 1), blood urea nitrogen (n = 2), uric acid (n = 4), glycated hemoglobin (n = 12), and urine protein-creatinine ratio (n = 1).

*P < 0.05 compared with low self-care score & low disease knowledge score; ^#^*P* < 0.05 compared with high self-care score and low disease knowledge score; ^&^*P* < 0.05 compared with low self-care score and high disease knowledge score.

### Determinants of self-care scale in patients with CKD

All variables in Table 1 were examined in univariate analysis linear regression analysis to find the determinants of self-care behavior (Table 2). Self-care behavior was positively correlated with disease knowledge, age, marital status, and duration of health education, but negatively correlated with smoking habit, currently working, receiving financial support from others, body mass index (BMI), and glycated hemoglobin level. We further put the above variables in multivariate forward analysis, which showed that high self-care scores were significantly associated with high disease knowledge scores, old age, being financially independent, not currently working, and having low glycated hemoglobin level.

**Table 2:** The determinant of self-care behavior and disease knowledge score in study subjects.

	Self-care behavior score				Disease knowledge score			
	Univariate		Multivariate (forward)		Univariate		Multivariate (forward)	
	β (95%Cl)	p-value	β (95%Cl)	p-value	β (95%Cl)	p-value	β (95%Cl)	p-value
**Clinical characteristics**								
Age (year)	0.16 (0.09, 0.23)	< 0.001	0.19 (0.11, 0.27)	< 0.001	− 0.14 (− 0.18, − 0.09)	< 0.001	− 0.12 (− 0.16, − 0.08)	< 0.001
Sex (male, %)	− 0.49 (− 2.30, 1.31)	0.6	–	–	− 0.61 (− 1.70, 0.40)	0.2	–	–
Smoking (yes, %)	− 2.20 (− 4.35, − 0.05)	0.04	–	–	− 0.99 (− 2.24, 0.26)	0.1	–	–
Alcohol consumption (yes, %)	− 0.78 (− 3.73, 2.15)	0.6	–	–	0.47 (− 1.23, 2.19)	0.6	–	–
Marital status (yes, %)	2.81 (0.71, 4.91)	0.009	–	–	1.20 (− 0.03, 2.43)	0.06	–	–
Currently working (yes, %)	− 3.53 (− 5.49, − 1.57)	< 0.001	− 3.97 (− 6.39, − 1.55)	0.001	2.46 (1.33, 3.59)	< 0.001	–	–
Independent finances (no,%)	− 2.11 (− 3.92, − 0.28)	0.02	− 3.26 (− 5.22, − 1.31)	0.001	− 2.63 (− 3.67, − 1.59)	< 0.001	–	–
Education (high school or above, %)	1.65 (− 0.14, 3.44)	0.07	–	–	4.22 (3.25, 5.19)	< 0.001	2.65 (1.69, 3.60)	< 0.001
Hypertension (yes, %)	1.87 (− 0.57, 4.32)	0.1	–	–	0.01 (− 1.42, 1.73)	0.9	–	–
Diabetes mellitus (yes, %)	− 1.62 (− 3.45, 0.22)	0.08	–	–	− 2.09 (− 3.15, − 1.03)	< 0.001	–	–
Heart disease (yes, %)	1.18 (− 1.09, 3.45)	0.3	–	–	− 1.16 (− 2.48, 0.16)	0.09	–	–
Body mass index (kg/m^2^)	− 0.37 (− 0.58, − 0.16)	< 0.001	–	–	− 0.15 (− 0.26, − 0.02)	0.02	–	–
Health education times	0.13 (0.04, 0.21)	0.004	–	–	0.09 (0.04, 0.14)	< 0.001	–	–
CKD duration (year)	0.08 (− 0.04, 0.19)	0.2	–	–	0.17 (0.10, 0.23)	< 0.001	0.13 (0.07, 0.19)	< 0.001
Blood urea nitrogen (mg/dl)	0.01 (− 0.03, 0.05)	0.6	–	–	0.01 (− 0.01, 0.04)	0.3	–	–
eGFR (ml/min/1.73 m^2^)	− 0.04 (− 0.08, 0.00)	0.07	–	–	0.00 (− 0.02, 0.02)	0.8	–	–
Log-formed glycated hemoglobin	− 0.17 (− 2.61, − 0.77)	< 0.001	− 1.13 (− 2.00, − 0.26)	0.01	− 1.24 (− 1.77, 0.71)	< 0.001	− 0.64 (− 1.11, − 0.17)	0.008
Hemoglobin (g/dl)	− 0.44 (− 0.89, 0.00)	0.05	–	–	0.13 (− 0.13, 0.39)	0.3	–	–
Albumin (g/dl)	1.27 (− 1.21, 3.74)	0.3	–	–	1.51 (0.08, 2.95)	0.04	–	–
Uric acid (mg/dl)	− 0.42 (− 0.99, 0.15)	0.1	–	–	− 0.12 (− 0.45, 0.22)	0.5	–	–
Cholesterol (mg/dl)	− 0.01 (− 0.03, 0.01)	0.5	–	–	0.01 (− 0.00, 0.02)	0.1	–	–
Log-formed triglyceride	− 5.05 (− 8.84, − 1.26)	0.009	–	–	− 1.63 (− 3.85, 0.58)	0.2	–	–
Log-formed urine protein/creatinine ratio	− 0.67 (− 2.23, 0.90)	0.4	–	–	− 0.54 (− 1.45, 0.37)	0.3	–	–
Self-care behavior	–	–	–	–	0.15 (0.10, 0.20)	< 0.001	0.15 (0.10, 0.20)	< 0.001
Disease knowledge	0.43 (0.28, 0.59)	< 0.001	0.51 (0.35, 0.66)	< 0.001	–	–		

CKD, chronic kidney disease; eGFR, estimated glomerular filtration rate.

### Determinants of disease knowledge in patients with CKD

After examining all variables in Table 1, we found that disease knowledge was positively correlated with self-care behavior, education level, currently working, duration of health education, duration of CKD, and serum albumin level, but negatively correlated with age, receiving financial support from others, history of diabetes, BMI, and glycated hemoglobin level in univariate linear regression analysis (Table 2). After adjusting the above variables in forward analysis, the results showed that high disease knowledge scores were significantly associated with high self-care behavior scores, young age, education level of high school or above, longer duration of CKD, and low glycated hemoglobin.

### Self-care behavior, disease knowledge, and rapid decrease in renal function in patients with CKD

The median eGFR slope of all patients was − 1.7 ml/min/1.73 m^2^ per year within 1 year before questionnaire examination (Table 3), and 147 patients (32.4%) had decline in eGFR > 3 ml/min/1.73 m^2^ per year. Both self-care behavior (odds ratio (OR): 0.97, 95% confidence index (CI): 0.95–0.99) and disease knowledge scores (OR: 0.93, 95% CI 0.89–0.96) were significantly associated with decline in eGFR > 3 ml/min/1.73 m^2^ per year in unadjusted analysis. After adjusting for age, sex, and all variables shown in Table 1 with p-value < 0.05, including DM, eGFR, log-formed urine protein-creatinine ratio (PCR), and hemoglobin level (Table S1), high self-care behavior (OR: 0.97, 95% CI 0.95–0.98) or high disease knowledge (OR: 0.91, 95% CI 0.87–0.95) scores were negatively and significantly associated with rapid decline in renal function. CKD patients in the highest quartile of self-care behavior or disease knowledge scores had lower risk of decline in eGFR > 3 ml/min/1.73 m^2^ per year compared to those in the lowest quartile. We also used forward regression analysis to investigate the association of rapid decline in renal function with self-care behavior and disease knowledge, and the results were still consistent (Table S2).

**Table 3:** The adjusted risks for rapid eGFR decline (eGFR decline more than 3 ml/min/1.73 m^2^/year) within 1 year prior to questionnaire examination in study patients according to self-care behavior and disease knowledge scores.

	eGFR slope	Proportion of eGFR decline more than 3 ml/min/1.73 m^2^/year	Rapid eGFR decline	p-value
	ml/min/1.73 m^2^/year		Adjusted odds ratio (95% Cl)	
**Self-care behavior (per score)**	− 1.7 (-4.0, 1.4)	147/454 (32.4)	0.97 (0.95–0.98)	0.002
**Self-care behavior (quartiles)**				
Quartile 1	− 2.4 (− 4.8, 1.3)	53/125 (42.4)	Reference	
Quartile 2	− 1.4 (− 4.2, 2.0)	37/107 (34.6)	0.73 (0.42–1.27)	0.3
Quartile 3	− 1.7 (− 3.4, 1.1)	35/126 (27.8)	0.55 (0.33–0.99)	0.04
Quartile 4	− 1.8 (− 2.9, 0.6)	22/96 (22.9)	0.38 (0.21–0.71)	0.002
**Disease knowledge (per score)**	− 1.7 (− 4.0, 1.4)	147/454 (32.4)	0.91 (0.87–0.95)	< 0.001
**Disease knowledge (quartiles)**				
Quartile 1	− 2.2 (− 5.3, 1.2)	59/138 (42.82)	Reference	
Quartile 2	− 2.1 (− 4.1, 1.4)	32/90 (35.6)	0.73 (0.41–1.31)	0.3
Quartile 3	− 1.5 (− 3.2, 0.9)	33/120 (27.5)	0.46 (0.26–0.80)	0.006
Quartile 4	− 1.5 (− 2.8, 2.3)	23/106 (21.7)	0.29 (0.15–0.54)	< 0.001
**Low self-care score and low disease knowledge score**	− 2.4 (− 5.0, 1.6)	58/133 (43.6)	Reference	
**High self-care score and low disease knowledge score**	− 2.1 (− 4.3, 1.2)	33/95 (34.7)	0.78 (0.44–1.38)	0.4
**Low self-care score and high disease knowledge score**	− 1.4 (− 4.0, 2.4)	32/99 (32.3)	0.58 (0.32–1.04)	0.07
**High self-care score and high disease knowledge score**	− 1.5 (− 2.7, 0.8)	24/127 (18.9)	0.28 (0.16–0.50)	< 0.001

Adjusted for age, sex and all variables in Table 1 whose p-value were < 0.05 including diabetes mellitus, hemoglobin, baseline estimated glomerular filtration rate, and log-formed urine protein-creatinine ratio in unadjusted model.

Self-care score quartile cut at 59, 67 and 72.

Disease knowledge score quartile cut at 18, 22 and 25.

Low self-care as less than median of self-care score; high self-care as above median of self-care score.

Low disease knowledge as less than median of disease knowledge score; high disease knowledge as above median of disease knowledge score.

There was also a significant interaction between self-care behavior and disease knowledge in rapid decline in renal function (p-interaction = 0.04). To investigate the effect of this interaction on rapid decline in renal function, we stratified CKD patients by the median score of self-care (67) and disease knowledge (22) respectively (Table 3). Patients with both high self-care and disease knowledge scores had decreased risk of rapid decline in renal function compared to those with both low self-care and disease knowledge scores.

In addition, the interaction between age and self-care score (p-interaction = 0.003) and that between age and disease knowledge score (p-interaction < 0.001) were found in our cohort. In order to examine the impact of age on the association of rapid decline in renal function with self-care behavior or disease knowledge, we stratified CKD patients according to the median of age with 67.0 years. The analysis showed significant dose dependent-responses in the relationships between rapid decline in renal function and self-care behavior score independent of age. Disease knowledge score was significantly associated with rapid decline in renal function only in CKD patients with age < 67 years (Table 4).

**Table 4:** The adjusted risks for rapid eGFR decline (eGFR decline > 3 ml/min/1.73 m^2^/year) within 1 year prior to questionnaire examination in study patients stratified by age according to self-care behavior and disease knowledge scores.

	Age ≥ median (67 years)			Age < median (67 years)		
	Percentage of eGFR decline > 3 ml/min/1.73 m^2^/year	Rapid eGFR decline		Percentage of eGFR decline > 3 ml/min/1.73 m^2^/year	Rapid eGFR decline	
		Adjusted odds ratio (95% Cl)	p-value		Adjusted odds ratio (95% Cl)	p-value
**Self-care behavior (per score)**	67/227 (29.5)	0.97 (0.94–1.00)	0.07	80/227 (35.2)	0.97 (0.94–0.99)	0.02
**Self-care behavior (quartiles)**						
Quartile 1	20/51 (39.2)	Reference		33/74 (44.6)	Reference	
Quartile 2	14/45 (31.1)	0.65 (0.27–1.55)	0.3	23/62 (37.1)	0.80 (0.39–1.66)	0.6
Quartile 3	22/76 (28.9)	0.66 (0.30–1.44)	0.3	13/50 (26.0)	0.48 (0.21–1.09)	0.07
Quartile 4	11/55 (20.0)	0.39 (0.16–0.93)	0.03	11/41 (26.8)	0.42 (0.17–1.00)	0.05
**Disease knowledge (per score)**	67/227 (29.5)	0.96 (0.90–1.01)	0.1	80/227 (35.2)	0.86 (0.81–0.92)	< 0.001
**Disease knowledge (quartiles)**						
Quartile 1	34/93 (36.6)	Reference		25/45 (55.6)	Reference	
Quartile 2	10/43 (23.3)	0.59 (0.25–1.36)	0.2	22/47 (46.8)	0.91 (0.37–2.22)	0.8
Quartile 3	15/56 (26.8)	0.69 (0.32–1.49)	0.4	18/64 (28.1)	0.30 (0.13–0.70)	0.006
Quartile 4	8/35 (22.9)	0.61 (0.24–1.55)	0.3	15/71 (21.1)	0.19 (0.08–0.46)	< 0.001
**Interaction between self-care score and disease knowledge score**	67/227 (29.5)	0.99 (0.99–1.00)	0.04	80/227 (35.2)	0.99 (0.99–0.99)	< 0.001
Low self-care score and low disease knowledge score	24/67 (35.8)	Reference		34/66 (51.5)	Reference	
High self-care score and low disease knowledge score	20/69 (29.0)	0.78 (0.37–1.64)	0.5	13/26 (50.0)	1.09 (0.41–2.86)	0.8
Low self-care score and high disease knowledge score	10/29 (34.5)	1.07 (0.41–2.80)	0.9	22/70 (31.4)	0.37 (0.17–0.78)	0.009
High self-care score and high disease knowledge score	13/62 (21.0)	0.55 (0.24–1.23)	0.1	11/65 (16.9)	0.16 (0.07–0.39)	< 0.001

Adjusted model of self-care behavior and disease knowledge in rapid eGFR decline included age, sex and all variables in Table 1 whose p-value were < 0.05 including diabetes mellitus, hemoglobin, baseline estimated glomerular filtration rate, and log-formed urine protein-creatinine ratio in unadjusted model.

Self-care score quartile cut at 59, 67 and 72.

Disease knowledge score quartile cut at 18, 22 and 25.

Low self-care as less than median of self-care score; high self-care as above median of self-care score.

Low disease knowledge as less than median of disease knowledge score; High disease knowledge as above median of disease knowledge score.

### Sensitivity analysis

We further analyzed the association of self-care behavior and disease knowledge with the decline in renal function within 2 years before enrollment. Both self-care behavior and disease knowledge were still significantly associated with rapid decline in renal function (Table S3), and significant interaction between these components in rapid decline in renal function (p-interaction = 0.02) was also shown.

## Discussion

To the best of our knowledge, this is the first study to evaluate the association of self-care behavior, disease knowledge and their interaction with the decline in renal function in patients with CKD enrolled in a multidisciplinary care program. The young CKD patients had better disease knowledge, while the older CKD patients had better self-care behavior. Self-care behavior was positively and significantly correlated with disease knowledge. Both high self-care behavior and disease knowledge scores were independently associated with reduced risk of rapid decline in renal function after adjusting for well-known risk factors. They also had an interaction effect on rapid decrease in renal function, and CKD patients with high scores for both had lower risk of rapid decline in renal function than those with low scores.

Our current results have supported a close association between self-care behavior and disease knowledge in patients with CKD^11^. Poor disease knowledge contributes to inadequate self-care behavior^12,13^, and both of them are barriers to efficient CKD care. Given the silent course of CKD, understanding the basic concepts about kidney function, symptoms of disease progression, and disease status is very important. Poor disease knowledge may cause difficulty in shared decision-making for the therapeutic strategy^12,13^. Therefore, to understand the determinants of disease knowledge and self-care behavior is very important for CKD care. Some socio-demographic factors (age, sex, marital status and education level), and socio-economic factors (occupation and financial support) have been known to be correlated with disease knowledge or self-care behavior^14,15^. In addition, some laboratory data, such as eGFR, is also correlated with disease knowledge or self-care behavior^14,15^. In our study, we found that CKD patients with both low self-care behavior and disease knowledge had higher proportion of DM than other groups, and glycated hemoglobin was significantly correlated with both these. Our results mean that CKD patients with good sugar control had great disease knowledge and self-care behavior, and this might be the cause or consequence of their performance.

On the other hand, we found that younger patients had higher scores of disease knowledge, while the older patients had better self-care behavior scores. The relationship between disease knowledge and rapid decline in renal function was only shown in CKD patients with age < 67 years, and the association of rapid decline in renal function with self-care behavior was independent of age. This is the first study to evaluate the interactional effect of age on the association of rapid decline in renal function with self-care behavior and disease knowledge in a CKD population. Aging is related to reduced functional capacity and multiple morbidities^16^, probably leading to insufficient disease knowledge in older patients with CKD. In addition, economic security, social participation and education are common problems in older patients^17^. Nevertheless, the awareness of poor physical function and multiple morbidities might drive older patients to have better self-care behavior. Although younger patients have high education level to gain better disease knowledge, they might not carry out correct behavior in self-care because of stress of work, not enough time, denial attitude, et al. Accumulating evidence shows that younger age is correlated with poor self-care behavior in chronic diseases, such as DM and heart failure. How to improve self-care behavior in this population needs more focus in the future^18,19^. Our findings might prompt the government and clinical staff to consider the effect of age when developing CKD care programs. Further research will be necessary to examine the relationship among physical function, comorbidity and self-care in patients with CKD.

Several care programs have been developed to help the general population and patients with CKD to learn about the potential risk factors for renal injury and the diagnosis of CKD, and to direct them to develop better self-care behavior^20,21^. Nevertheless, it is still not easy to reach the goals of these programs. Gray et al. assessed disease knowledge in CKD patients after 1 year of educational interventions, and found that their disease knowledge remained limited with only small changes compared to baseline^22^. Yen et al. also reported that educational intervention increased disease knowledge at 6 months but that it had dropped again by 12 months^23^. However, we found that increased duration of multidisciplinary CKD care was positively correlated with high self-care behavior scores, which further reduced the risk of rapid decline in renal function. CKD patients might attain more disease knowledge and achieve better self-care behavior through increasing the frequency of educational interventions. Through understanding the level of self-care behavior and disease knowledge, multidisciplinary care teams could develop precise and individualized CKD care program based on age to further improve the efficiency of CKD care.

Schrauben et al. reported that different phenotypes of self-care behavior were associated with clinical outcomes in patients with CKD^24^. They used blood pressure, smoking, BMI, diet habit and physical activity to categorize the types of self-care behavior, and found that CKD patients with phenotype III self-care behavior, including smoking, uncontrolled blood pressure, poor diet, and physical inactivity, had high risk of adverse clinical outcomes. In this study, we used a structured questionnaire, which has been validated in Taiwanese with CKD^14^, to access self-care behavior in our patients with CKD. Apart from the similar domains of measurements of self-care behavior by Schrauben et al^24^, the novel finding of our questionnaires is considering medication adherence in the evaluation of self-care behavior (Table S4). High score of medication adherence (data not shown) was significantly associated with decreased risk of rapid decline in renal function in our CKD patients. Good medication adherence has been proven to reduce the risk of hospitalization, morbidity and all-cause mortality in different populations^25,26^. The Chronic Renal Insufficiency Cohort Study also supported the association of low medication adherence with increased risk of CKD progression^27^. Our findings provide complete and various aspects to investigate the relationship between self-care behavior and the decline in renal function in CKD.

There are several limitations to this study. Firstly, we used the questionnaire to examine self-care behavior and disease knowledge in CKD patients, and recall bias may have affected the results. We excluded patients unable to complete the questionnaires by themselves. Thus, the results may not be applied to all populations. Nevertheless, we believe that the questionnaire is a useful and convenient tool to evaluate self-care behavior and disease knowledge in patients with CKD for clinical staff to provide immediate suggestions necessary to meet the patients’ needs. Secondly, we only measured self-care behavior and disease knowledge once at enrollment, and the effect of changes in these components over time on the decline in renal function could not be evaluated. Besides, this study retrospectively examines the association of the interaction between self-care behavior and disease knowledge with decline in renal function. Further prospective study is necessary to evaluate the impact of their dynamic change on renal progression. Finally, we only enrolled CKD patients in the outpatient department, and the result may not be applied to all CKD population.

In conclusion, we demonstrated significant association of self-care behavior, disease knowledge and their interaction with rapid decline in renal function in patients with CKD. Enhancing self-care behavior and disease knowledge might reverse or alleviate decline in renal function. These findings could be used to improve the decline in renal function and establish CKD care program models.

## Materials and methods

### Study participants

**Figure 1:**
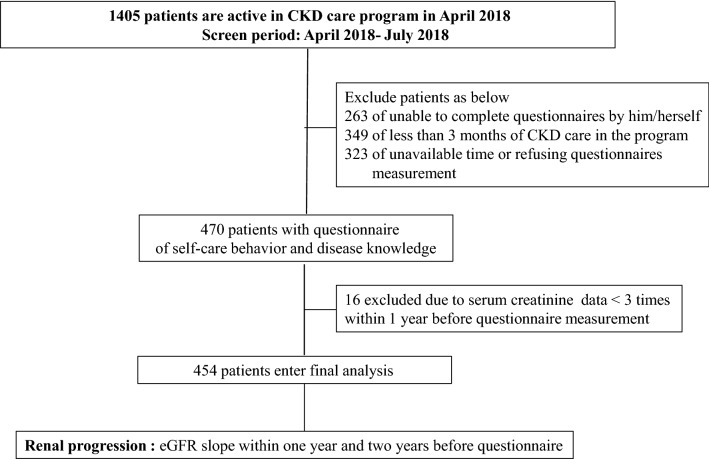
The flow chart of study subjects enrollment.

This retrospective study included patients with CKD stages 1–5 not on renal replacement therapy, who have been enrolled in the integrated CKD program at Kaohsiung Medical University Hospital (KMUH), a tertiary hospital in southern Taiwan. The CKD program is to improve quality-of-care, and is managed by a cross-disciplinary team, including clinical physicians, nursing staff members, nutritionists, and pharmacists. More than 6000 patients have been included in the CKD program from November 2003 to April 2018. During nearly 15 years, 4595 patients seceded from CKD program due to commencing dialysis (n = 1704), transplantation (n = 13), death (n = 469), and transferring to other clinics (n = 1675), refusing long-term health care (n = 217) or loss of follow-up (n = 517). Thus, 1405 patients were still active in the program in April 2018. All of 1405 patients had been scheduled for regular outpatient visits between April 2018 and July 2018, and were invited and included for study interview. Among these patients, 263 unable to complete the questionnaires by themselves or to care for themselves in daily life, 349 having less than 3 months in CKD care program, and 323 having unavailable time or refusing to enter this study were excluded. Besides, 16 of less than 3 eGFR measurements 1 year prior to the study interview were also excluded for estimating eGFR slope precisely. Finally, 454 participants entered final analysis (Fig. 1).

The definition of CKD was staged based on Kidney Disease Outcomes Quality Initiative (K/DOQI) guidelines and eGFR (ml/min/1.73 m^2^) was calculated using the equation of the 4-variable Modification of Diet in Renal Disease (MDRD) Study equation (stage 1 ≥ 90; stage 2 60–89; stage 3a 45–59; stage 3b 30–44; stage 4 15–29; stage 5 < 15)^28,29^. There were no significant differences in age and CKD stage between patients who did and did not enter the study (Table S5). The study protocol was approved by the Institutional Review Board of KMUH. Informed consent was obtained in written form from all patients and all clinical investigations were conducted according to the principles expressed in the Declaration of Helsinki.

### Clinical measurements

Information of socio-demographic characteristics and clinical data including age, sex, tobacco smoking (yes *v.s.* no), alcohol consumption (yes *v.s.* no), marital status (married *v.s* single, divorced or widowed), education level (senior high school or above *v.s*. junior high school or below), current occupation (currently working *v.s.* no job or retirement), financial support (by self *v.s.* from others), and co-morbidities such as DM, hypertension, and heart disease, were obtained from medical records at KMUH and interviews with patients at enrollment of this study. DM was defined as a medical history of the disease, the use of anti-diabetic drugs, or blood glucose values defined in the American Diabetes Association criteria. Patients with a history of hypertension, taking antihypertensive drugs, or blood pressure measurements ≥ 140/90 mmHg were defined as having hypertension. Heart disease was defined as medical history of myocardial infarction, ischemic heart disease or congestive heart failure. The duration of CKD and the duration of CKD care were calculated from self-reports and enrollment in the multidisciplinary CKD care program respectively. The number of health and nutrition education sessions were calculated between enrollment into the multidisciplinary CKD care program and study interview. Information regarding the patients’ medications, including angiotensin converting enzyme inhibitor (ACEI) and angiotensin II receptor blocker (ARB) was recorded. The use of ACEI/ARB was defined as having long-term prescription (at least 3 ys) for these medications before the study at the study interview. Blood samples were taken after a 12-h fast for biochemistry studies, and urine protein levels were measured using urine PCR before the study interview.

### Self-care behavior and disease knowledge measurement

The participants completed structured questionnaires of self-care behavior and disease knowledge, which were developed for patients with CKD, at study enrollment. We used the CKD Self-Care (CKDSC) scale to measure self-care behavior. CKDSC is 16-item questionnaire with total scores (score range (SR): 16–80) and five subscales including medication adherence (SR: 5–25), diet control (SR: 4–20), exercise (SR: 3–15), smoking behaviors (SR: 2–10), and blood pressure monitoring (SR: 2–10), which has been validated for use in patients with CKD^14^.

Kidney Knowledge Survey (KIKS), which includes 34 questions related to general knowledge, knowledge of kidney function and knowledge of symptoms of progression or failure, is known as a reliable tool to measure objective knowledge of kidney disease in patients with CKD^30^. Furthermore, Wright et al. developed a perceived kidney knowledge survey (PIKS, SR: 9–36), which included nine questions (medications that help the kidney, medications that can hurt the kidney, foods to avoid if kidney function is low, blood pressure goal, treatment options if kidney function gets worse, symptoms of CKD, how kidney function is checked, functions of the kidney, why patient was sent to a kidney doctor), to understand perceived kidney knowledge more, make earlier presentations to the clinic and improve clinical communication in CKD cohort^15^. Thus, we used PIKS to measure disease knowledge in our patients with CKD.

### The measurement of the decline in renal function

We defined the outcome as the decline in renal function. The decline in renal function was evaluated according to the eGFR slope, which was defined as the regression coefficient between eGFR and time and presented as ml/min/1.73 m^2^ per year. All eGFR values within the 2 years prior to completing the questionnaire of self-care behavior and disease knowledge were collected, with at least three eGFR values being required to estimate the eGFR slope. Rapid decline in renal function was defined as the eGFR decline > 3 ml/min/1.73 m^2^ per year^31^.

### Statistical analysis

The study participants were classified in to four groups based on the median scores of both self-care behavior and disease knowledge. Multiple imputation-expectation maximization was utilized to handle missing data. The data were expressed as mean ± SD or median (25th, 75th percentile) for continuous variables, and percentages for categorical variables. One-way analysis of variance (ANOVA) followed by the post hoc test adjusted with a Bonferroni correction or the Kruskal–Wallis H test was utilized for among-group comparisons. The chi-square test was used to evaluate differences in the distribution of categorical variables. Linear regression analysis was utilized to identify the factors related to self-care behavior and disease knowledge. All variables in Table 1 with p-value < 0.05 in univariate linear regression analysis were further put into forward linear regression analysis. We used multivariate logistic regression to examine the association of rapid decline in renal function within 1 year prior to the questionnaire with self-care behavior and disease knowledge firstly, and then evaluated the association of rapid decline in renal function within 2 years prior to the questionnaire with self-care behavior and disease knowledge as sensitivity analysis. Multivariate models were adjusted for age, sex, and all variables in Table 1 with p-value < 0.05 in the univariate analysis including DM, hemoglobin, baseline eGFR, and log-formed urine PCR. Forward analysis with adjusting all variables in Table 1 was also utilized to diminish selecting bias of statistical analysis. The interaction among age, self-care behavior and disease knowledge was tested in rapid decline in renal function using logistic regression analysis. Statistical analyses were conducted using SPSS version 18.0 for Windows (SPSS Inc., Chicago, Illinois). Statistical significance was set at a two-sided p-value of ≤ 0.05.

## Supplementary Information

Supplementary Information.
