# Supplementary material for: The interaction between self-care behavior and disease knowledge on the decline in renal function in chronic kidney disease
Source: Sci Rep. 2021 Jan 11;11:401. doi: 10.1038/s41598-020-79873-z (PMC7801646; doi:10.1038/s41598-020-79873-z)
Supplement: Supplementary file 1 — Supplementary Information. [file 41598_2020_79873_MOESM1_ESM.pdf]

The interaction between self-care behavior and disease knowledge on the decline in renal function in chronic kidney disease

Yi-Chun Tsai[1-5#], Shu-Li Wang[6#], Hui-Ju Tsai[7], Tzu-Hui Chen[6], Lan-Fang Kung[6], Pei-Ni Hsiao[6], Shih-Ming Hsiao[6], Shang-Jyh Hwang[1,3,4], Hung-Chun Chen[1,3,4], Yi-Wen Chiu[1,3,4*]

[1]Department of Nephrology, Department of Internal Medicine, Kaohsiung Medical University Hospital, Kaohsiung Medical University, Kaohsiung, Taiwan

[2]Division of General Medicine, Department of Internal Medicine, Kaohsiung Medical University Hospital, Kaohsiung Medical University, Kaohsiung, Taiwan

[3]Faculty of Renal Care, Kaohsiung Medical University, Kaohsiung, Taiwan

[4]School of Medicine, College of Medicine, Kaohsiung Medical University, Kaohsiung, Taiwan

[5]Cohort Research Center, Kaohsiung Medical University, Kaohsiung, Taiwan.

[6]Department of Nursing, Kaohsiung Medical University Hospital, Kaohsiung Medical University

[7]Department of Family Medicine, Kaohsiung Municipal Ta-Tung Hospital, Kaohsiung Medical University Hospital, Kaohsiung Medical University, Kaohsiung, Taiwan

Table S1. The risk for rapid eGFR for rapid eGFR decline (eGFR decline more than 3ml/min/1.73m^2/year) within one year prior to questionnaire examination in study patients in univariate logistic analysis

	Univariate OR(95%Cl)	p-value
**Clinical characteristics**		
Age (year)	0.99(0.97-1.00)	0.1
Sex (male, %)	1.10(0.74-1.62)	0.6
Smoking (yes, %)	1.35(0.85-2.14)	0.2
Alcohol consumption (yes, %)	0.53(0.26-1.10)	0.1
Hypertension (yes, %)	1.41(0.80-2.47)	0.2
Diabetes mellitus (yes, %)	1.55(1.03-2.30)	0.03
Heart disease (yes, %)	1.20(0.73-1.95)	0.4
ACEI/ARB (yes, %)	0.79(0.52-1.22)	0.3
Marital status (yes, %)	0.91(0.57-1.44)	0.7
Currently working (yes, %)	1.21(0.79-1.86)	0.4
Independent finances (yes, %)	1.20(0.81-1.79)	0.4
Education (high school or above, %)	0.93(0.63-1.39)	0.7
Body mass index (kg/m^2)	1.01(0.96-1.05)	0.8
Blood urea nitrogen (mg/dl)	1.01(0.99-1.01)	0.3
eGFR (ml/min/1.73m^2)	0.99(0.98-0.99)	0.02
Log-formed glycated hemoglobin	1.11(0.91-1.35)	0.3
Hemoglobin (g/dl)	0.87(0.79-0.96)	0.008
Albumin (g/dl)	0.82(0.48-1.40)	0.5
Uric acid (mg/dl)	1.05(0.93-1.19)	0.4
Cholesterol (mg/dl)	1.00(0.99-1.01)	0.3
Log-formed triglyceride	1.59(0.69-3.65)	0.3
Log-formed urine protein/creatinine ratio	1.84(1.29-2.62)	0.01
Self-care behavior (per score)	0.97(0.95-0.99)	0.001
Disease knowledge (per score)	0.93(0.89-0.96)	<0.001

Table S2. The adjusted risks for rapid eGFR decline (eGFR decline more than 3ml/min/1.73m^2/year) within one year prior to questionnaire examination in study patients according to self-care behavior and disease knowledge scores using forward analysis

	Rapid eGFR decline	
	Adjusted Odds ratio (95% Cl)	p-value
**Self-care behavior (per score)**	0.97(0.95-0.99)	0.003
**Self-care behavior (Quartiles)**		
Quartile 1	Reference	
Quartile 2	0.74(0.43-1.28)	0.3
Quartile 3	0.57(0.33-0.98)	0.04
Quartile 4	0.42(0.30-0.77)	0.005
**Disease knowledge (per score)**	0.92(0.88-0.95)	<0.001
**Disease knowledge (Quartiles)**		
Quartile 1	Reference	
Quartile 2	0.73(0.41-1.29)	0.3
Quartile 3	0.48(0.27-0.83)	0.009
Quartile 4	0.32(0.17-0.59)	<0.001
**Low self-care score & low disease knowledge score**	Reference	
**High self-care score & low disease knowledge score**	0.76(0.43-1.33)	0.3
**Low self-care score & high disease knowledge score**	0.68(0.39-1.19)	0.2
**High self-care score & high disease knowledge score**	0.33(0.19-0.58)	<0.001

Forward analysis was adjusted for all variables in Table 1

Self-care score quartile cut at 59, 67 and 72.

Disease knowledge score quartile cut at 18, 22 and 25

Low self-care as less than median of self-care score; High self-care as above median of self-care score

Low disease knowledge as less than median of disease knowledge score; High disease knowledge as above median of disease knowledge score

Table S3. The adjusted risks for rapid eGFR decline (eGFR decline more than 3ml/min/1.73m^2/year) within two years prior to questionnaire examination in study patients according to self-care behavior and disease knowledge scores

	eGFR slope ml/min/1.73m^2/year	Portion of eGFR decline more than 3ml/min/1.73m^2/year	Rapid eGFR decline Adjusted Odds ratio (95% Cl)	p-value
**Self-care behavior (per score)**	-2.0 (-4.3,0.0)	167/454 (36.8)	0.97(0.95-0.99)	0.005
**Self-care behavior (Quartiles)**				
Quartile 1	-2.5(-5.3,-0.5)	58/125 (46.4)	Reference	
Quartile 2	-2.0(-4.0,0.3)	39/107 (36.4)	0.73(0.42-1.27)	0.3
Quartile 3	-1.7(-4.3,0.3)	42/126 (33.3)	0.60(0.35-1.04)	0.1
Quartile 4	-2.0(-3.6,0.1)	28/96 (29.2)	0.47(0.26-0.85)	0.01
**Disease knowledge (per score)**	-1.8(-1.2,0.1)	167/454 (36.8)	0.95(0.91-0.98)	0.006
**Disease knowledge (Quartiles)**				
Quartile 1	-2.2(-4.9,-0.0)	61/138 (44.2)	Reference	
Quartile 2	-2.1(-4.4,0.3)	31/90 (34.4)	0.73(0.41-1.32)	0.3
Quartile 3	-1.9(-3.9,-0.1)	44/120 (36.7)	0.76(0.44-1.31)	0.3
Quartile 4	-1.6(-3.8,0.1)	31/106 (29.2)	0.43(0.23-0.79)	0.007
Low self-care score & low disease knowledge score	-2.3(-5.1,-0.3)	59/133 (44.4)	Reference	
High self-care score & low disease knowledge score	-2.0(-4.5,0.7)	33/95 (34.7)	0.75(0.42-1.32)	0.3
Low self-care score & high disease knowledge score	-2.2(-4.3,-0.0)	38/99 (38.4)	0.85(0.48-1.50)	0.5
High self-care score & high disease knowledge score	-1.6(-3.5,0.0)	37/127 (29.1)	0.48(0.28-0.83)	0.01

Adjusted for age, sex and all variables in Table 1 whose p-value were <0.05 (age, sex, diabetes mellitus, hemoglobin, baseline estimated glomerular filtration rate, and log-formed urine protein-creatinine ratio) in unadjusted model.

Self-care score quartile cut at 59, 67 and 72.

Disease knowledge score quartile cut at 18, 22 and 25

Table S4. The distribution of five subscales of self-care behavior in study patients stratified by the medians levels of self-care behavior and disease knowledge

Self-care	Entire Cohort N=454	Low self-care score & low disease knowledge score N=133	High self-care score & low disease knowledge score N=95	Low self-care score & high disease knowledge score N=99	High self-care score & high disease knowledge score N=127	p-value
Total scores	64.1±9.7	55.3±7.0$^{\#\&}$	71.6±3.9$^{*\&}$	58.0±6.7$^{*\#}$	72.5±4.0$^{*\&}$	<0.001
Medication adherence	23.5±2.8	22.5±3.2$^{\#}$	24.5±1.3$^{*\&}$	22.5±3.7$^{\#}$	24.5±1.2$^{*\&}$	<0.001
Diet control	14.6±.3.5	12.0±3.0$^{\#\&}$	16.6±2.5$^{*\&}$	12.9±2.8$^{*\#}$	17.1±2.4$^{*\&}$	<0.001
Exercise	10.0±4.2	6.4±3.2$^{\#\&}$	13.4±2.1$^{*\&}$	7.8±3.4$^{*\#}$	13.1±2.3$^{*\&}$	<0.001
Smoking behavior	9.1±2.1	8.8±2.3$^{\#}$	9.7±1.0$^{*\&}$	8.2±2.9$^{\#}$	9.5±1.5$^{*\&}$	<0.001
Blood pressure monitoring	6.8±2.6	5.7±2.5$^{\#}$	7.3±2.6*	6.4±2.5	7.9±2.3$^{*\&}$	<0.001

Data are expressed as number (percentage) for categorical variables and mean±SD

Low self-care as less than median of self-care score (67); High self-care as above median of self-care score (67)

Low disease knowledge as less than median of disease knowledge score (22); High disease knowledge as above median of disease knowledge score (22)

$^{*}P < 0.05$ compared with low self-care score & low disease knowledge score; $^{\#}P < 0.05$ compared with high self-care score & low disease knowledge score; $^{\&}P < 0.05$ compared with low self-care score & high disease knowledge score

Table S5. Distributions of sex, age, and chronic kidney disease (CKD) stages between subjects with questionnaire measurement and those without questionnaire measurement

	**Cohort of CKD care program (n = 1405)**	**Questionnaire group (n = 470)**	**Non-questionnaire group (n = 935)**	**p-value**
Age (years)				
< 60	24.8	26.2	24.2	0.4
≥ 60	75.2	73.8	75.8	
Sex				0.02
male	60.1	55.7	62.2	
Female	39.9	44.3	37.8	
CKD stage				0.1
1-3a	25.0	25.7	24.6	
3b	24.1	20.4	26.0	
4	27.3	28.3	26.8	
5	23.6	25.5	22.6	

Stage 1: eGFR ≥ 90; stage 2: eGFR 60-89; stage 3a: eGFR 45-59; stage 3b: eGFR 30-44; stage 4: eGFR 15-29; stage 5: eGFR < 15 ml/min/1.73m^2
